# Pressure-Induced Assembly of Organic Phase-Change Materials Hybridized with Expanded Graphite and Carbon Nanotubes for Direct Solar Thermal Harvesting and Thermoelectric Conversion

**DOI:** 10.3390/nano14242047

**Published:** 2024-12-21

**Authors:** Jie Ji, Yizhe Liu, Xiaoxiang Li, Yangzhe Xu, Ting Hu, Zhengzheng Li, Peng Tao, Tao Deng

**Affiliations:** 1 State Key Laboratory of Metal Matrix Composites, School of Materials Science and Engineering, Shanghai Jiao Tong University, 800 Dong Chuan Road, Shanghai 200240, China; 2 Carbon Materials Research Institute, Baowu Carbon Technology Co., Ltd., 1800 Tongji Road, Baoshan District, Shanghai 201999, China; 3 National Engineering Research Center of Special Equipment and Power System for Ship and Marine Engineering, Shanghai 200030, China

**Keywords:** phase-change material, expanded graphite, carbon nanotube, compression molding, thermal conductivity

## Abstract

Direct harvesting of abundant solar thermal energy within organic phase-change materials (PCMs) has emerged as a promising way to overcome the intermittency of renewable solar energy and pursue high-efficiency heating-related applications. Organic PCMs, however, generally suffer from several common shortcomings including melting-induced leakage, poor solar absorption, and low thermal conductivity. Compounding organic PCMs with single-component carbon materials faces the difficulty in achieving optimized comprehensive performance enhancement. Herein, this work reports the employment of hybrid expanded graphite (EG) and carbon nanotubes (CNTs) to simultaneously realize leakage-proofness, high solar absorptance, high thermal conductivity, and large latent heat storage capacity. The PCM composites were prepared by directly mixing commercial high-temperature paraffin (HPA) powders, EG, and CNTs, followed by subsequent mechanical compression molding. The HPA-EG composites loaded with 20 wt% of EG could effectively suppress melting-induced leakage. After further compounding with 1 wt% of CNTs, the form-stable HPA-EG20-CNT1 composites achieved an axial and in-plane thermal conductivity of 4.15 W/m K and 18.22 W/m K, and a melting enthalpy of 165.4 J/g, respectively. Through increasing the loading of CNTs to 10 wt% in the top thin layer, we further prepared double-layer HPA-EG-CNT composites, which have a high surface solar absorptance of 92.9% for the direct conversion of concentrated solar illumination into storable latent heat. The charged composites could be combined with a thermoelectric generator to release the stored latent heat and generate electricity, which could power up small electric devices such as light-emitting diodes. This work demonstrates the potential for employing hybrid fillers to optimize the thermophysical properties and solar thermal harvesting performances of organic PCMs.

## 1. Introduction

Sustainable development of human society urges the utilization of clean energy to reduce environmental pollution and reduce the dependence on non-renewable fossil fuels [1,2,3]. Converting renewable energy into thermal energy for storage and subsequent use in heating-related applications can overcome the intermittency of many renewable energy sources and address the mismatch between heating supply and consumption need [4]. In recent years, solar thermal conversion has gained increasing attention due to its cleanness, high efficiency, abundance, cost advantages, and easy accessibility [5,6]. Organic solid–liquid phase-change materials (PCMs) such as paraffin wax have emerged as the favorite substances to store solar thermal energy as latent heat owing to their large latent heat storage capacity, tunable charging/discharging temperature, chemical abundance, consistent reversible phase-change behavior, and good thermal and chemical stabilities [7,8,9]. Meanwhile, organic PCMs suffer from an inherent low light-to-thermal conversion ability, melting-induced leakage, and low thermal conductivity that affects the charging/discharging rates [10,11,12].

To overcome these shortcomings, previous research efforts were focused on compounding organic PCMs with other functional fillers to prepare phase-change composites. For instance, microcapsules [13,14], porous materials [15,16,17,18], and organic copolymers [19,20,21,22,23,24] have been employed to confine the melted PCMs within restricted space and avoid the flow of liquid PCMs during the solid–liquid phase-change processes [25]. Although microencapsulation and compounding with organic copolymers could effectively suppress melting leakage, the resultant composites still suffer from low thermal conductivity and poor solar absorption [13,26]. By contrast, impregnating organic PCMs within low-density porous carbon materials, such as graphene aerogels [27,28,29], graphite foams [30,31,32], biomass carbonized foams [33,34,35], carbon nanotube (CNT) arrays [36,37], carbon fibers [38,39], and expanded graphite (EG) [40,41,42,43,44], can simultaneously improve the anti-leakage performance, solar absorptance, and effective thermal conductivity of PCM composites [45]. In these carbon-enhanced composites, the connected carbon networks can simultaneously provide the capillary confinement effect to maintain shape stability, the skeleton to trap incident solar photons, and the conduction path to timely transfer heat during the charging/discharging process as well. In general, the higher the loading of the carbon fillers, the stronger the enhancement of the thermophysical properties including the leakage-proofness, solar absorptance, and thermal conductivity. The high loading of carbon fillers, however, reduces the effective latent heat capacity of the composites, and adds extra cost to the resultant phase-change composites. The massive loading of expensive carbon fillers such as graphene, CNTs, and carbon fibers limits the scalable fabrication and application of PCM composites at low cost [46,47,48,49].

In comparison, EG has been considered as a promising filler to explore the large-scale preparation of PCM composites owing to its unique worm-like structure, high thermal conductivity, and cost-effectiveness [50]. In recent years, EG had been compounded with PCM powders and mechanically compressed into tightly packed composites for scalable thermal energy harvesting and thermal management applications [40,41,42,43]. The pressed composite blocks, however, often possess smooth reflective surfaces because of the horizontal alignment of graphite sheets under mechanical compression [50]. Moreover, the coating of an organic PCM layer on the surface of assembled graphite sheets hinders heat transfer between neighboring sheets and thus limits further enhancement of the effective thermal conductivity of the composites [40,41,42,43]. To date, achieving optimal performances of PCM composites including leakage-proofness, high solar absorptance, large latent heat capacity, and high thermal conductivity with a low carbon filler loading remains a grand challenge.

**Figure 1:**
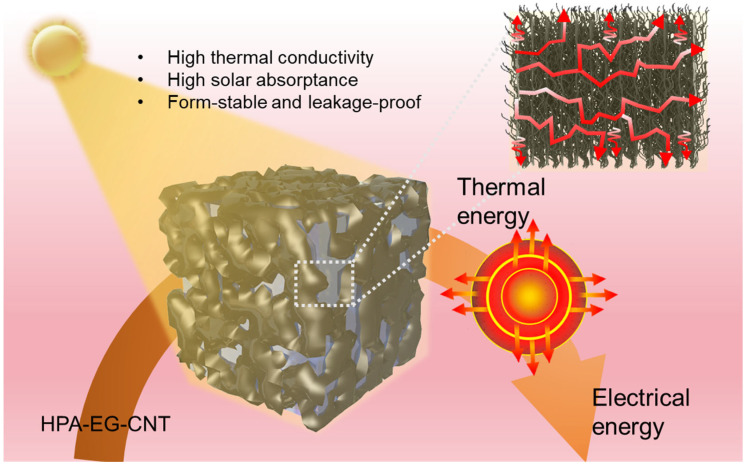
Pressure-induced assembly of organic PCM hybridized with EG and CNT for high-performance direct solar thermal energy harvesting and thermoelectric conversion.

In this work, we reported the design and fabrication of organic PCM composites through the direct powder mixing of commercial high-temperature paraffin (HPA), EG, and CNTs followed by mechanical compression to optimize the comprehensive improvement of the thermophysical properties for direct solar thermal energy harvesting and thermoelectric conversion (Figure 1). The worm-like porous yet connected structure of EG (20 wt%) not only enables the adsorption of the melted PCMs and prevention of leakage but also forms three-dimensional thermal conduction networks after compression-induced assembly to enhance the effective thermal conductivity of the PCM composites. The addition of a minute amount of CNTs not only significantly increased the solar absorptance but also formed the heat conduction bridges between neighboring graphite sheets. Finally, we prepared double-layer PCM composites for the high-performance direct harvesting of solar thermal energy and thermoelectric conversion. The thin top layer has a high concentration of CNTs (10 wt%) to efficiently harvest solar thermal energy. The main bulk HPA composite, which is loaded with 20 wt% of EG and 1 wt% of CNT, achieved an axial and in-plane thermal conductivity of 4.15 W/m K and 18.22 W/m K, respectively. We demonstrated that such double-layer HPA-EG-CNT composites could rapidly convert concentrated solar illumination into high-temperature storable latent heat. Through coupling with a thermoelectric generator (TEG), the latent heat could be released to generate electricity with a high output voltage of more than 3 V within the phase-change temperature range, which could power up a light-emitting diode (LED) bulb.

## 2. Materials and Methods

### 2.1. Materials

HPA powders were purchased from Dongguan Zhangmutou Suzhan Plastics Business Department (Dongguan, China), with a powder size of 1000 mesh. EG powder and CNTs were purchased from Suqian Xigu Nano Technology Co., Ltd (Suqian, China). The CNTs had an average length of 8 to 20 μm, an outer diameter of 12.9 ± 3.5 nm, and a layer number of 8 to 15.

### 2.2. Preparation of Phase-Change Composites

In a typical process of HPA-EG-CNT composites, EG, CNT, and HPA powders were mixed in a specific mass ratio and uniformly blended using a mixer (MX-S, Dragon Lab, Beijing, China). The mixture was then heated in a drying oven (GZX-9076MBE, Shanghai Boxun Medical Biological Instrument Corp., Shanghai, China) at 125 °C for 2 h, allowing the HPA to melt and adhere to the surface of the EG sheets. After cooling, the mixture was placed into a mold and subjected to compression by an electric press (YLJ-40TA, Hefei Kejing Materials Technology Co., Ltd., Hefei, China) with a pressure of ~8 Pa for two min to obtain samples with a defined shape. The HPA-EG PCM composites were prepared by using the same method. The mass fraction range of EG in the HPA-EG PCM composites was 5–20%, and the mass fraction range of CNT in the HPA-EG-CNT composites was 0.5–2%. Detailed compositions for all the prepared samples are listed in Table 1.

### 2.3. Characterization

The microstructure of the EG and PCM composites was characterized by using a scanning electron microscope (SEM, RISE-MAGNA, TESCAN, Brno, Czech Republic). The phase-change enthalpy and phase-change temperature of the HPA, HPA-EG, and HPA-EG-CNT composites were characterized by a differential scanning calorimeter (DSC 2500, TA, New castle, DE, USA), with a heating and cooling rate of 10 °C/min. A laser flash thermal analyzer (LFA 467, NETZCH, Selb, Germany) was used to measure the in-plane and axial (through-plane) thermal conductivity of the PCM composites as a function of the content of EG and CNT. The absorption spectra of the PCM composites were measured by a UV-Vis-NIR spectrophotometer (Lamda 950, Perkin Elmer, Shelton, CT, USA).

### 2.4. Solar Thermal Conversion and Storage Test

The samples were placed under a xenon lamp with adjustable illumination power density (GHX-Xe-300, Shanghai Bilon Instrument Co., Ltd., Shanghai, China) to simulate the solar heating process. The intensity of the simulated light was measured by a light power meter (CEL-NP2000, Beijing China Education Au-light Co. Ltd., Beijing, China). The temperature at the bottom of the samples was measured by a K-type thermocouple. During the heating process, the thermal imaging of the sample surface was recorded by an infrared camera (FLIR T640, FLIR, Wilsonville, OR, USA).

### 2.5. Thermoelectric Conversion Experiment

The pre-heated PCM composites (25 mm × 25 mm × 3 mm) and a cold plate at −5 °C were connected to the hot and the cold side of the TEG (TEHP1-12730H, Beijing Yonghao Weiye Technology Co., Ltd., Beijing, China) to create the temperature gradient to drive thermoelectric conversion. The commercial TEG module (TEHP1-12730H) has a dimension of 30 mm × 30 mm × 3.65 mm and an AC resistance@23 °C of 3.8 Ω. When the hot side temperature is 220 °C and the cold side temperature is 30 °C, the open-circuit voltage is 7.9 V and the matched load output voltage is 3.9 V. The output voltage was measured by a data acquisition instrument (34972A, KEYSIGHT, Santa Rosa, CA, USA). The commercial LED bulbs we used have a red color and a suggested working voltage range of 1.8 to 2.2 V.

## 3. Results and Discussion

### 3.1. Fabrication and Characterization of HPA-EG and HPA-EG-CNT Composites

**Figure 2:**
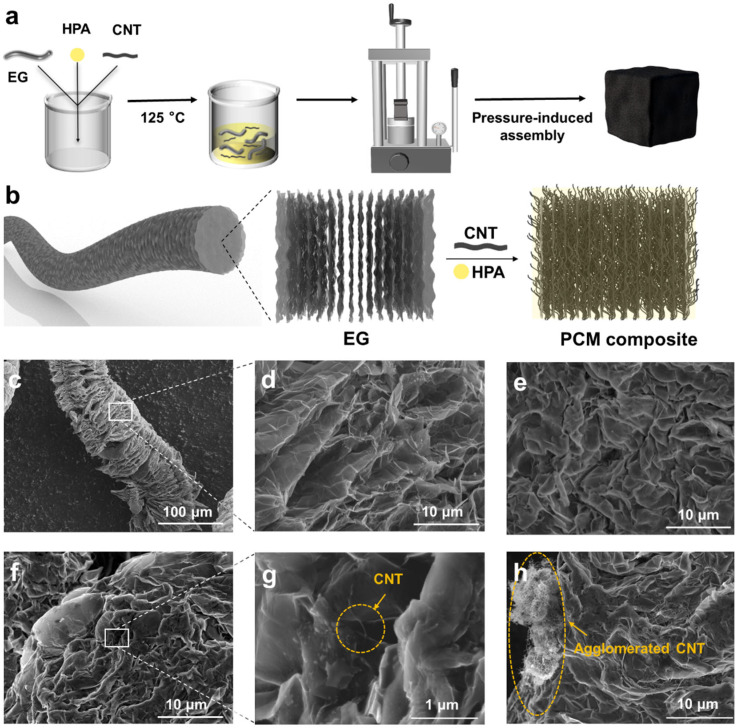
(**a**) Schematic showing the preparation process of PCM composites through pressure-induced assembly. (**b**) Schematic structure of PCM composites. (**c**,**d**) SEM images of EG at low and high magnification. (**e**) SEM image showing adsorption of 20 wt% of HPA within EG after heating treatment. (**f**,**g**) SEM image of HPA-EG20-CNT1 composites at low and high magnification. (**h**) SEM image showing agglomeration of CNTs within the HPA-EG20-CNT2 composites.

As schemed by Figure 2a, the PCM composites were prepared through blending EG powders, HPA powders, and CNTs and melting the mixture at 125 °C for 2 h before being subjected to mechanical compression within a mold. The porous structure of the EG powders allowed for the facile incorporation of the HPA PCM powders and CNT in between the connected graphite sheets. After the melting treatment, the HPA and CNTs were coated onto the surface of the graphite sheets, and the soaked EG powders were then mechanically compressed into tight compacts (Figure 2b). The SEM images in Figure 2c,d present that the EG powders are composed of porous yet connected graphite sheets, which provide the capability to form conductive networks and ample space to load PCMs. SEM observation indicated that the HPA powders and CNTs were uniformly distributed within the porous space provided by the EG (Figure S1), and the added HPA was quickly adsorbed onto the surface of the graphite sheets after the heating treatment (Figure 2e). Figure 2f shows that after compression molding, the randomly distributed EG sheets formed an anisotropic layered structure and the whole HPA-EG-CNT composites became tightly packed. At high magnifications, Figure 2g shows the uniform decoration of CNTs on the surfaces of the graphite sheets within the HPA-EG20-CNT1 composites (Figure S1). However, when the loading of CNTs increased to 2 wt%, obvious agglomeration was observed within the composites (Figure 2h).

### 3.2. Thermophysical Properties of HPA-EG-CNT Composites

**Figure 3:**
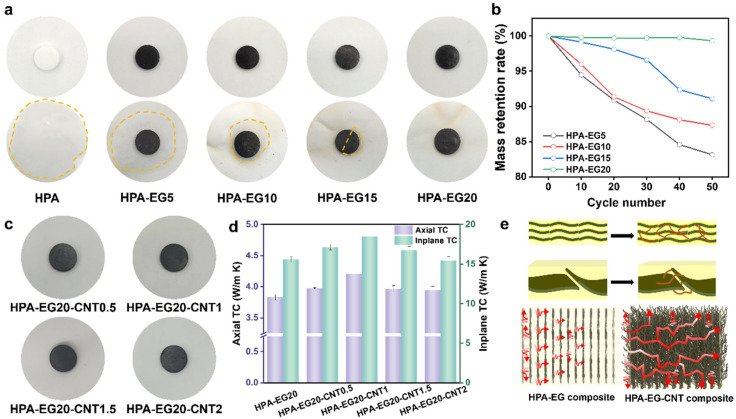
(**a**) Photographs comparing anti-leakage performance of HPA and HPA-EG composites with different loadings of EG. (**b**) Mass retention rate of HPA-EG composites during leakage tests. (**c**) Photographs showing leakage-proofness of HPA-EG20-CNT composites. (**d**) Axial and in-plane thermal conductivity of HPA-EG and HPA-EG-CNT composites. (**e**) Schematic showing enhancement of heat conduction of HPA-EG composites through further compounding with CNTs.

Melting-induced leakage has been viewed as an obstacle impeding the facile application of solid–liquid PCMs. Herein, the porous 3D structure of the assembled EG networks provides the capillary confinement effect that limits the leakage of melted HPA and the suppressing of leakage is dependent on the loading of EG. Figure 3a shows that after heating at 130 °C for 1 h, the neat HPA spreads and wets the filter paper beneath. In comparison, the leakage of the melted HPA from the HPA-EG composites is effectively suppressed with the increasing loading of the EG powders. When the EG loading reached 20 wt%, no leakage of the melted HPA was observed for the HPA-EG20 composites. To quantitatively analyze the anti-leakage performance, we monitored the mass change in the HPA-EG composites during cycled heating/cooling tests. Figure 3b confirms that the HPA-EG20 composites have negligible mass loss, and the mass loss becomes smaller for the HPA-EG composites with the increasing loading of the EG powders. Figure 3c shows that further compounding the HPA-EG20 composites with different loadings of CNTs (0.5 wt%, 1 wt%, 1.5 wt%, and 2 wt%) did not affect their anti-leakage performances.

The EG powders not only afforded the composites with strong resistance to melting-induced leakage but also significantly improved the effective thermal conductivity. We compared the thermal conductivity of the compression-molded HPA-EG and HPA-EG-CNT composites with pristine HPA in both the in-plane and axial directions. It was found that the effective thermal conductivity of the HPA-EG composites along the axial direction gradually increases from 2.58 W/m K to 3.75 W/m K when the loading of the EG powders increases from 5 wt% to 15 wt% (Figure S2), which is several times that of the thermal conductivity of HPA (~0.4 W/m K). Figure 3d presents that the HPA-EG20 composites have an in-plane and axial thermal conductivity of 15.58 W/m K and 3.83 W/m K, respectively. Such anisotropic thermal conductivity is related to the orientated distribution of graphite plates within the compressed composites. After compounding with CNTs, Figure 3d shows that the in-plane and axial thermal conductivity of the composites was further enhanced, but the improvement is dependent on the loading of CNTs. With 1 wt% loading of CNTs, the in-plane and axial thermal conductivity reached a maximum value of 18.22 W/m K and 4.15 W/m K, respectively, which is 16.7% and 8.3% higher than that of the HPA-EG20 composites. The enhancement effect was weakened when the loading of CNTs was further increased to 1.5 wt% and 2 wt%. As schemed by Figure 3e, without the addition of the CNTs, the heat transfer between neighboring graphite sheets is limited by the coating layer of HPA, which has a low thermal conductivity. Within the HPA-EG-CNT composites, the added high-thermal-conductivity CNTs can serve as thermal conduction bridges between neighboring graphite sheets. In this case, they form the graphite–CNT–graphite heat transfer path, which is superior to the graphite–HPA–graphite path for heat conduction in both the in-plane and axial directions. The observed weakened enhancement effect in high-loading composites (1.5 wt%, 2 wt%) is related to the formation of agglomerated CNTs, which broke the thermal connection path (Figure S3) [51].

**Figure 4:**
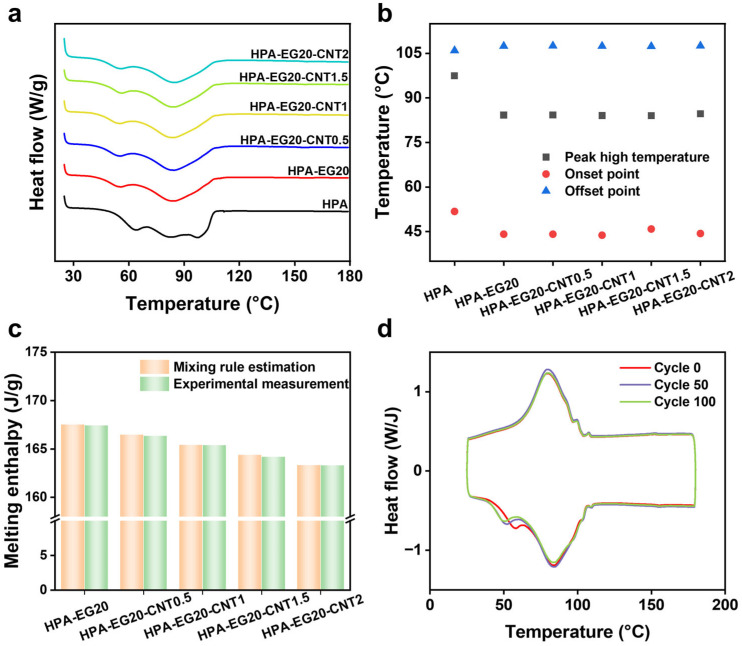
(**a**) Endothermic DSC curves of HPA, HPA-EG20, and HPA-EG20-CNT composites. (**b**) Peak, onset, and offset melting temperature of HPA, HPA-EG20, and HPA-EG20-CNT composites. (**c**) Melting enthalpy of HPA, HPA-EG20, and HPA-EG20-CNT composites. (**d**) DSC curves of HPA-EG20-CNT1 composites before and after cycled melting/solidification.

The endothermic DSC curve of neat HPA in Figure 4a shows broad heat-absorbing peaks ranging from 52 °C to 106 °C, which could be related to the mixed alkane chain lengths of paraffin in commercial products. In comparison with neat HPA, the endothermic peak temperature and the melting onset temperature of the HPA-EG20 and HPA-EG20-CNT composites have a noticeable shift from 97 °C and 52 °C to around 84 °C and 45 °C, respectively (Figure 4b). The melting offset temperature remains largely constant without a noticeable change. The enhanced thermal conductivity enables a fast thermal response and promotes the occurrence of solid–liquid phase change within the composites in both charging and discharging processes (Figure S4).

Figure 4c presents that the melting enthalpy of HPA-EG20 and HPA-EG20-CNT composites gradually decreases with the increasing loading of EG and CNT, and the general trend can be estimated by the rule of mixture. The melting enthalpy of the HPA-EG20-CNT1 composite that has the highest thermal conductivity has a melting enthalpy of 165.4 J/g. As shown by the nearly overlapped DSC curves in Figure 4d, the HPA-EG20-CNT1 composites have demonstrated consistent solid–liquid phase-change behavior when they were subjected to 100 cycles of heating and cooling between 25 °C and 130 °C. In addition, the HPA-EG20-CNT1 also exhibited good mechanical robustness under thermal cycling after loading 500 g of weight (Figure S5), and maintained the mechanical integrity after 100 thermal cycles (Figure S6). The observed mechanical robustness of the composites should be attributed to the tight compact and connected structure of the composites prepared by the pressure-induced compression molding approach.

**Figure 5:**
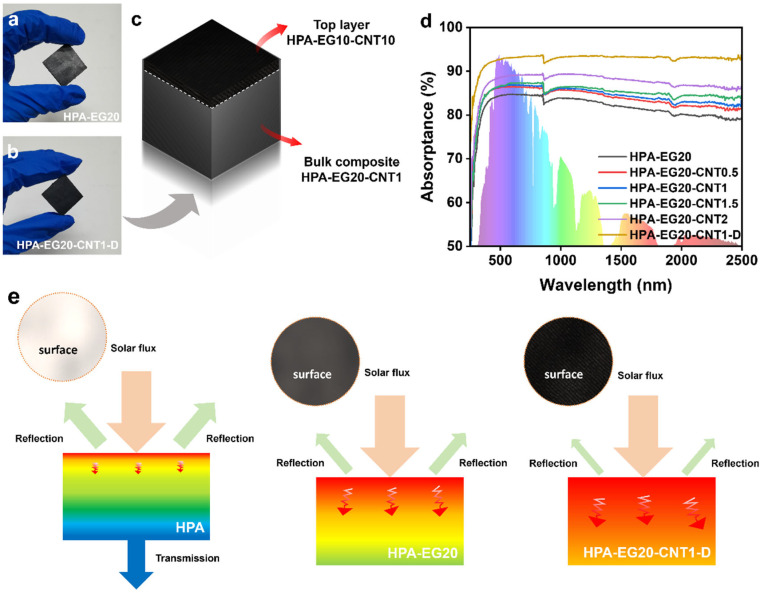
(**a**) Photograph of HPA-EG20 composites showing gray surfaces. (**b**) Photograph of double-layer structure HPA-EG20-CNT1-D composites. (**c**) Schematic structure of HPA-EG20-CNT1-D composites. (**d**) Optical absorption spectra of HPA-EG and HPA-EG-CNT composites. (**e**) Schematics comparing solar absorption by HPA, HPA-EG20, and HPA-EG20-CNT1-D composites.

Neat HPA PCMs show a whitish appearance and have a solar absorptance lower than 10% in the visible-light region (Figure S7). Although both EG and CNT can efficiently absorb broadband sunlight [52,53], Figure 5a shows that the HPA-EG20 composites demonstrate grayish surface appearance, while the HPA-EG20-CNT sample shows a deeply dark surface (Figure 5b). To simultaneously achieve high surface solar absorptance and high thermal conductivity, we prepared double-layer PCM composites (Figure 5c) that have 10 wt% of CNT and 10 wt% of EG on the top thin layer (thickness: ~0.1 mm) and employ HPA-EG20-CNT1 as the bulk thermal storage material (HPA-EG20-CNT1-D).

The absorption spectra in Figure 5d show that the solar absorptance of the HPA-EG20 composites reached 83.7%. After further compounding with CNT, the HPA-EG20-CNT composites showed higher solar absorptance with the increasing loading of CNTs. When the loading of CNT reached 2 wt%, the HPA-EG20-CNT2 composite achieved 88.6% absorption of sunlight. The double-layer HPA-EG20-CNT1-D composites have shown a high surface solar absorptance of 92.96%. As schemed in Figure 5e, the whitish translucent HPA has low solar absorptance due to the lack of solar-absorbing components and partial transmission loss (Figure S8). In the HPA-EG20-CNT1-D composites, the reduced EG content and high loading of CNTs contribute to increasing surface roughness, which can efficiently trap incident solar photons and reduce surface reflection loss.

### 3.3. Direct Solar Thermal Harvesting Performance

**Figure 6:**
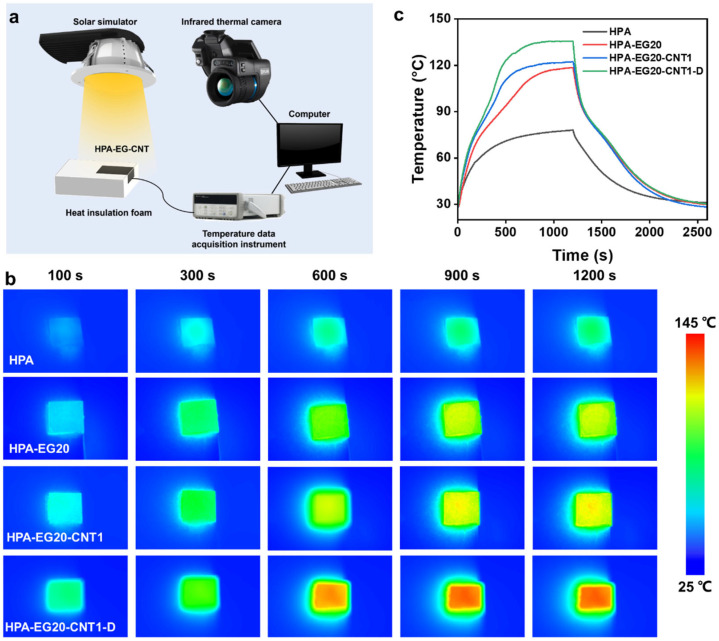
(**a**) Schematic of experimental setup for evaluating direct solar thermal harvesting performance. (**b**) Time-sequential infrared images showing charging process of HPA, HPA-EG20, and HPA-EG20-CNT composites. (**c**) Temperature evolution profiles during solar thermal charging and natural cooling discharging processes.

To evaluate the solar thermal harvesting performance of the HPA-EG20-CNT1-D composites, the sample was placed in an insulating melamine foam and irradiated by simulated sunlight with a power density of 8 kW/m^2^ (Figure 6a). The temperature change in the sample was simultaneously recorded by placing a thermocouple at the bottom of the sample (thickness: 2 mm) and using an infrared camera to monitor the surface temperature change from the top view. Time-sequential infrared images in Figure 6b and temperature evolution profiles in Figure 6c present that all samples reached an equilibrium temperature after being subjected to direct concentrated solar illumination for 20 min. Due to its poor light absorption and low thermal conductivity, the HPA sample had a slow thermal response and the bottom of the HPA sample only reached a temperature of 77 °C after solar illumination for 20 min, which could not induce complete melting of HPA.

Under the same charging conditions, the HPA-EG20 composites have shown a much faster temperature rise than the neat HPA and were heated to 118 °C. Such an equilibrium temperature is higher than the offset melting temperature of HPA, implying that the PCM was fully melted. Obvious deflection of the temperature curves in the temperature range of 75–110 °C also indicates the occurrence of solid–liquid phase change. In comparison, the temperature rise in the HPA-EG20-CNT1 sample was much faster than the HPA-EG20 sample and the bottom equilibrium temperature reached 122 °C. It took ~400 s for the HPA-EG20-CNT1 sample to complete the solid–liquid phase change, which is shorter than the HPA-EG20 sample (734 s). By contrast, the HPA-EG20-CNT1-D composites have shown the optimal heating speed and equilibrium temperature, with a bottom equilibrium temperature reaching 135 °C, which is much higher than the offset melting temperature.

The low solar absorption rate of pure HPA leads to poor solar thermal energy collection, and its low thermal conductivity cannot timely transfer the heat absorbed by the surface to the interior of the sample, resulting in serious heat loss. The high loading of CNTs in the top layer of the HPA-EG20-CNT1-D can effectively absorb sunlight and convert it into thermal energy, while the main body has high thermal conductivity, which can quickly conduct the solar thermal energy to all parts of the PCM composite and store it as latent heat. After the simulated sunlight is turned off, the temperature of the PCM composite first drops rapidly, then releases latent heat when it reaches the solidification temperature range, with the solidification process lasting for ~400 s, and then continues to release sensible heat until it reaches room temperature. The high thermal conductivity of the PCM composites enables rapid uniform solar thermal heating and natural cooling during the charging and discharging processes.

To evaluate the cycled charging and discharging performance of the HPA-EG20-CNT1-D composites, a small-sized sample (12.5 mm × 12.5 mm × 1 mm) was subjected to continuous concentrated solar illumination and natural cooling for 10 cycles. As shown in Figure S9, the heating and cooling temperature curves remined largely the same in terms of the maximum heating temperature and heating and cooling rates during the cycled tests, confirming the good cycling stability of the HPA-EG20-CNT1-D composites under direct concentrated solar illumination (8 kW/m^2^ and 5 kW/m^2^).

### 3.4. Thermoelectric Conversion

**Figure 7:**
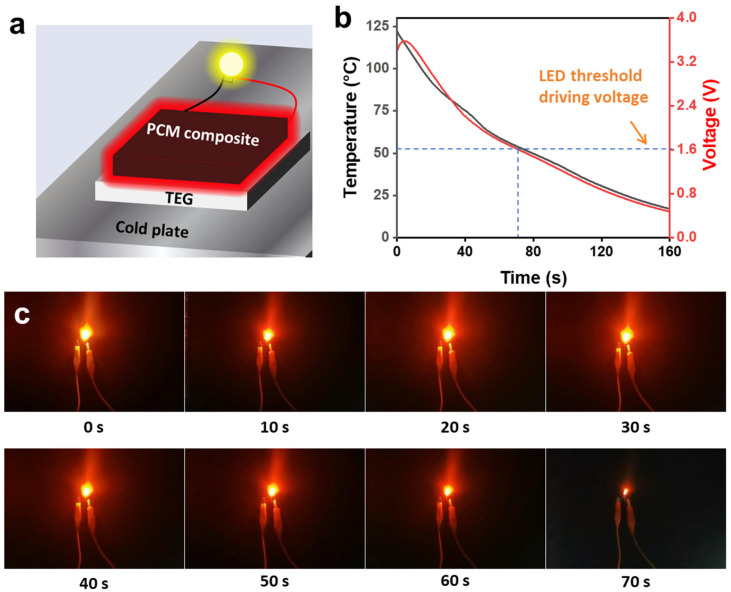
(**a**) Schematic experimental setup for releasing solar thermal energy stored within HPA-EG20-CNT1-D composites and thermoelectric conversion. (**b**) Temperature evolution profile and corresponding output voltage during discharging. (**c**) Time-sequential photographs showing lighting up an LED bulb with generated electrical output.

The high melting temperature of the HPA can provide a large temperature gradient for high-performance thermoelectric conversion. As shown in Figure 7a, the HPA-EG20-CNT1-D (25 mm × 25 mm × 3 mm), which was fully charged to 135 °C, was placed on the hot side of the thermoelectric generator (TEG), and a temperature-controlled cold plate (−5 °C) was connected to the cold side of the TEG. The electrical output was used to drive an LED bulb that has an activation voltage of 1.6 V. During the experiment, the maximum output voltage of the TEG could reach approximately 3.6 V (Figure 7b). When the PCM begins to solidify at a temperature of ~106 °C, the output voltage is ~3.4 V, and when the solidification is complete, the output voltage is ~1.5 V.

During the discharging process, Figure 7c presents that the electrical output could light up the LED bulb for ~70 s with high brightness (Video S1). We also conducted a control experiment by using conventional paraffin that has a phase-change temperature of 52 °C to construct the PCM composites. During the phase-change process, the output voltage was ~1.7 V (Figure S10), and the LED bulb was lit for only about 20 s, emitting very weak light (Video S2).

## 4. Conclusions

In summary, this work demonstrated that compounding organic PCMs with hybrid EG and CNTs could achieve the optimal comprehensive enhancement of thermophysical properties including resistance to melting-induced leakage, solar absorptance, thermal conductivity, and latent heat storage capacity. The worm-like porous structure of EG enables massive loading of the PCM without leakage occurring while providing connected heat transfer networks after compression molding. The minute amount of CNTs not only serves as the heat conduction bridge between compressed EG sheets but also enhances the solar absorptance of the PCM composites. The powder blending and mechanical compression molding approach allow for compounding PCMs with different functional carbon fillers into tightly connected leakage-proof composite blocks. The hybrid filler design and tailorable filler concentration offer extra means to optimize the thermophysical properties and functionalities of the PCM composites. We demonstrated that such a hybrid filler design and compression molding approach for the fabrication of double-layer PCM composites can efficiently harvest solar thermal energy for high-performance thermoelectric conversion. It is anticipated that the hybridized filler design and generally applicable fabrication process could be employed to prepare high-performance PCM composites for a variety of heating-related applications, and the fillers can be other low-cost porous carbon such as carbonized biochar beyond EG.

## Figures and Tables

**Table 1:** Composition of phase-change composites.

Sample	HPA (wt%)	EG (wt%)	CNT (wt%)
HPA	100	0	0
HPA-EG5	95	5	0
HPA-EG10	90	10	0
HPA-EG15	85	15	0
HPA-EG20	80	20	0
HPA-EG20-CNT0.5	79.5	20	0.5
HPA-EG20-CNT1	79	20	1
HPA-EG20-CNT1.5	78.5	20	1.5
HPA-EG20-CNT2	78	20	2

## Data Availability

The original contributions presented in the study are included in the article, further inquiries can be directed to the corresponding author.

## Supplementary Materials

The following supporting information can be downloaded at: https://www.mdpi.com/article/10.3390/nano14242047/s1, Figure S1: (a) SEM image showing homogeneous loading of HPA powders within porous EG networks. (b) SEM image showing uniform distribution of HPA powders and CNTs within porous EG. (c) SEM image showing impregnation of HPA and CNT within EG after heating treatment. (d) SEM image of HPA-EG20-CNT1 composites at high magnification; Figure S2: axial thermal conductivity of HPA-EG composites loaded with different concentration of EG (5 wt%, 10 wt%, 15 wt%, 20 wt%); Figure S3: SEM images showing the distribution of CNTs within the PCM composites: (a) HPA-EG20-CNT0.5; (b) HPA-EG20-CNT1; (c) HPA-EG20-CNT1.5; (d) HPA-EG20-CNT2; Figure S4: exothermic DSC curves of HPA, HPA-EG20, and HPA-EG20-CNT composites; Figure S5: photograph and infrared image showing placing 500 g of weight on HPA-EG20-CNT1 during the phase transition process when it is heated by a hot plate at 130 °C; Figure S6: photograph showing mechanical integrity and compression resistance of HPA-EG20-CNT1 composite after 100 thermal cycles; Figure S7: absorption spectrum of neat HPA; Figure S8: transmission spectra of HPA, HPA-EG20, and HPA-EG20-CNT1-D composites; Figure S9: recycled charging and discharging tests by placing the HPA-EG20-CNT1-D composites under direct concentrated solar illumination (a: 8 kW/m^2^, b: 5 kW/m^2^) followed by natural cooling; Figure S10: temperature evolution profile and corresponding output voltage during discharging of PA-EG20 composites; Video S1: discharging of HPA-EG20-CNT1-D composites for driving thermoelectric conversion and lighting up an LED bulb; Video S2: discharging of PA-EG20-CNT1-D composites for driving thermoelectric conversion and lighting up an LED bulb.
