# Supplementary material for: Pressure-Induced Assembly of Organic Phase-Change Materials Hybridized with Expanded Graphite and Carbon Nanotubes for Direct Solar Thermal Harvesting and Thermoelectric Conversion
Source: Nanomaterials (Basel). 2024 Dec 21;14(24):2047. doi: 10.3390/nano14242047 (PMC11678627; doi:10.3390/nano14242047)
Supplement: Supplementary file 1 [file nanomaterials-14-02047-s001.zip › nanomaterials-3362539-supplementary.pdf]

Pressure-Induced Assembly of Organic Phase-Change
Materials Hybridized with Expanded Graphite and Carbon Nanotubes for Direct Solar Thermal Harvesting and
Thermoelectric Conversion

Jie Ji [1], Yizhe Liu [1], Xiaoxiang Li [1], Yangzhe Xu [1], Ting Hu [1], Zhengzheng Li [2,*], Peng Tao [1,3,*] and Tao Deng [1,3]

[1] State Key Laboratory of Metal Matrix Composites, School of Materials Science and Engineering, Shanghai Jiao Tong University, 800 Dong Chuan Road, Shanghai 200240, China; sea_son@sjtu.edu.cn (J.J.); 780145898@sjtu.edu.cn (Y.L.); lixiaoxiang@sjtu.edu.cn (X.L.); xuyz1998@sjtu.edu.cn (Y.X.); ht-huting@sjtu.edu.cn (T.H.); dengtao@sjtu.edu.cn (T.D.)
[2] Carbon Materials Research Institute, Baowu Carbon Technology Co., Ltd., 1800 Tongji Road, Baoshan District, Shanghai 201999, China
[3] National Engineering Research Center of Special Equipment and Power System for Ship and Marine Engineering, Shanghai 200030, China
* Correspondence: lizhengzheng@baosteel.com (Z.L.); taopeng@sjtu.edu.cn (P.T.)

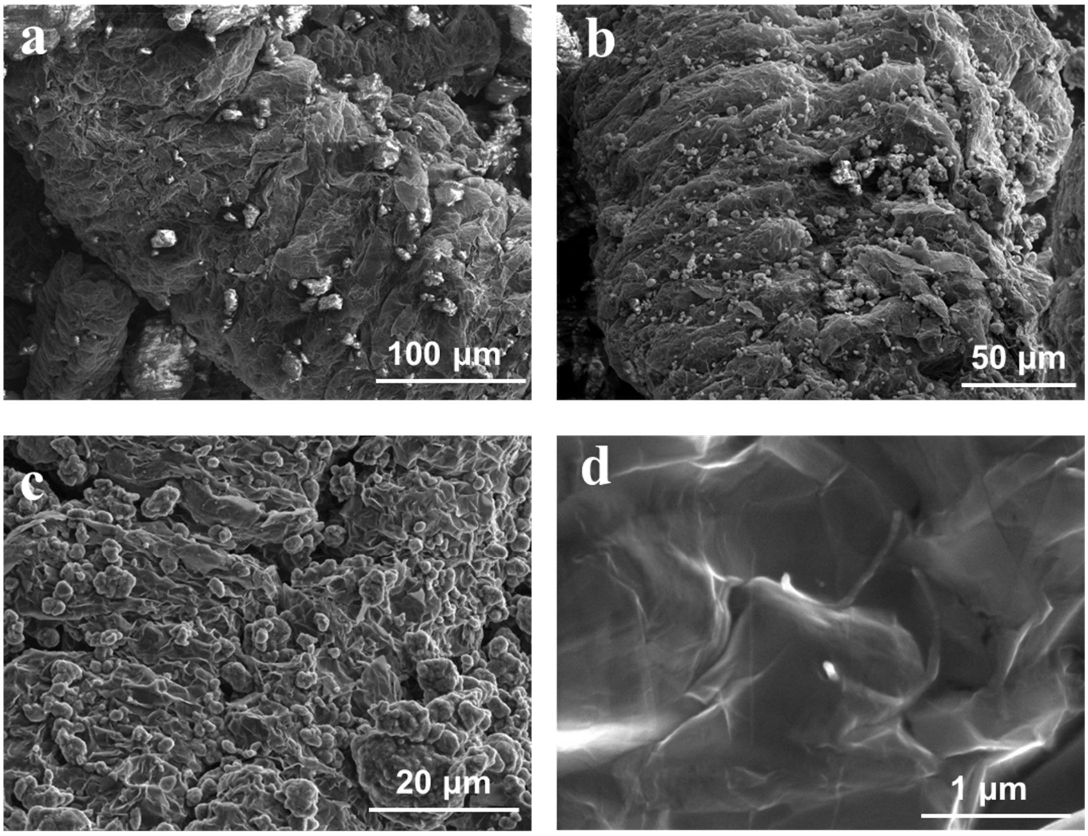

Figure S1. (a) SEM image showing homogeneous loading of HPA powders within porous EG networks. (b) SEM image showing uniform distribution of HPA powders and CNTs within porous EG. (c) SEM image showing impregnation of HPA and CNT within EG after heating treatment. (d) SEM image of HPA-EG20-CNT1 composites at high magnification.

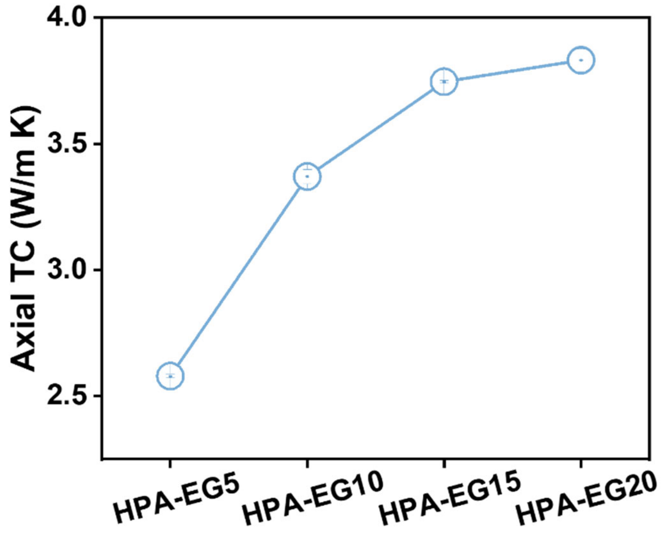

Figure S2. Axial thermal conductivity of HPA-EG composites loaded with different concentration of EG (5 wt%, 10 wt%, 15 wt%, 20 wt%).

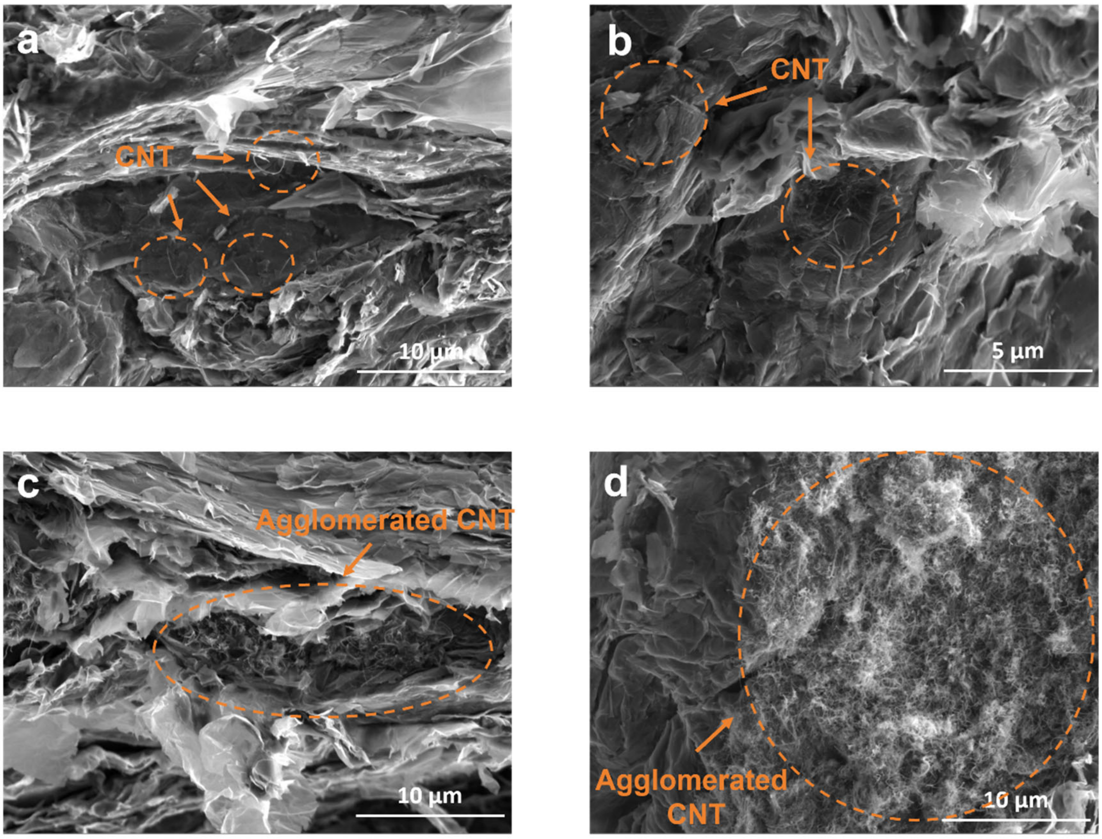

Figure S3. SEM images showing the distribution of CNTs within PCM composites: (a) HPA-EG20-CNT0.5; (b) HPA-EG20-CNT1; (c) HPA-EG20-CNT1.5; (d) HPA-EG20-CNT2.

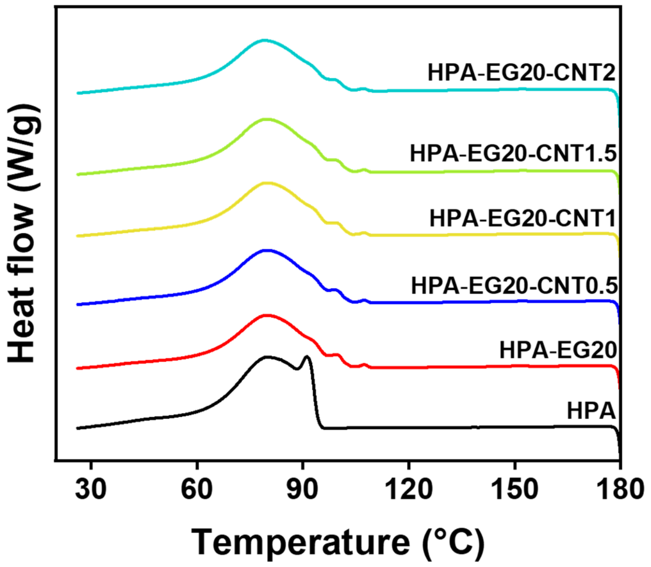

Figure S4. Exothermic DSC curves of HPA, HPA-EG20 and HPA-EG20-CNT composites.

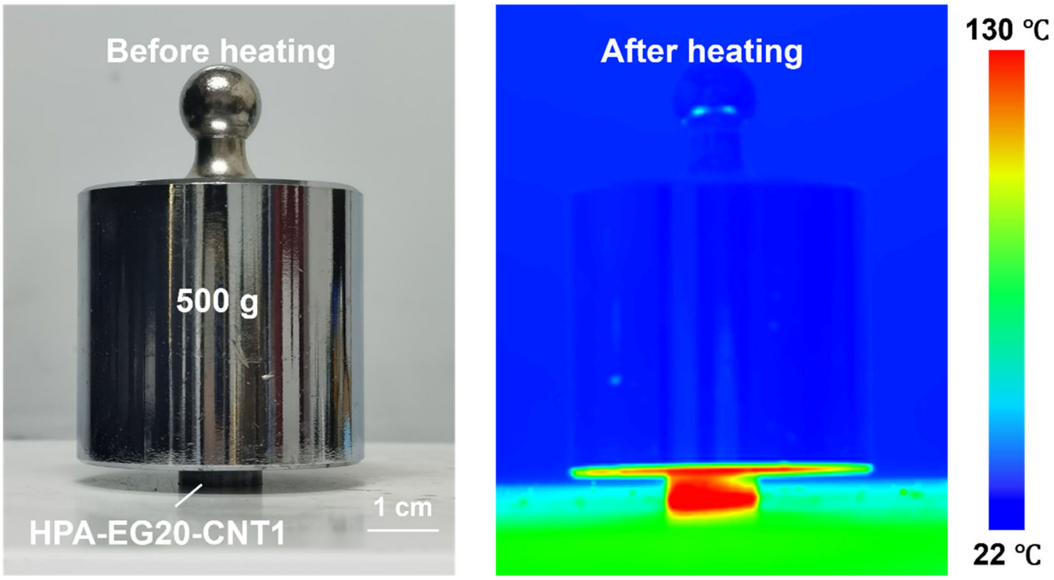

Figure S5. Photograph and infrared image showing placing 500 g of weight on HPA-EG20-CNT1 during the phase transition process when it is heated by a hot plate at 130 ℃.

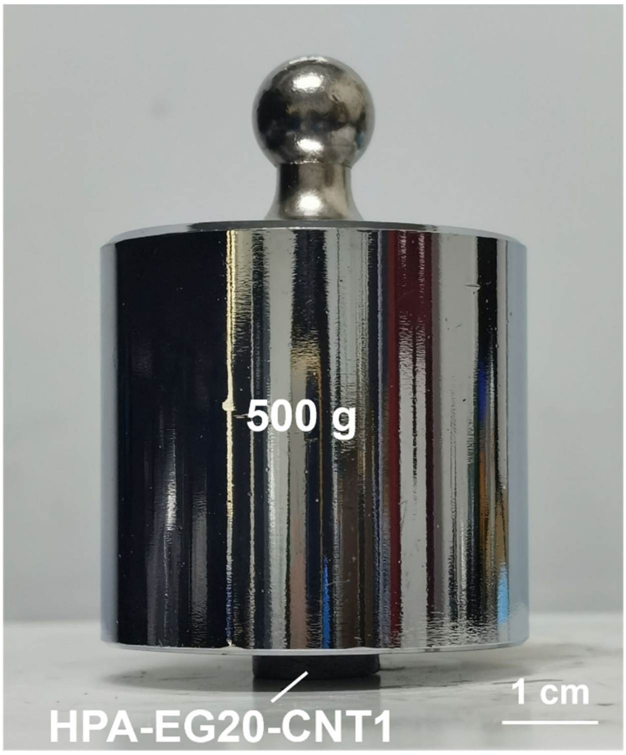

Figure S6. Photograph showing mechanical integrity and compression resistance of HPA-EG20-CNT1 composite after 100 thermal cycles.

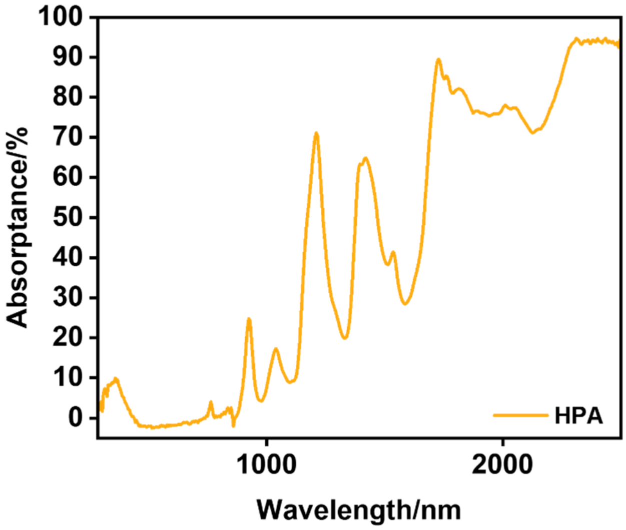

Figure S7. Absorption spectrum of neat HPA.

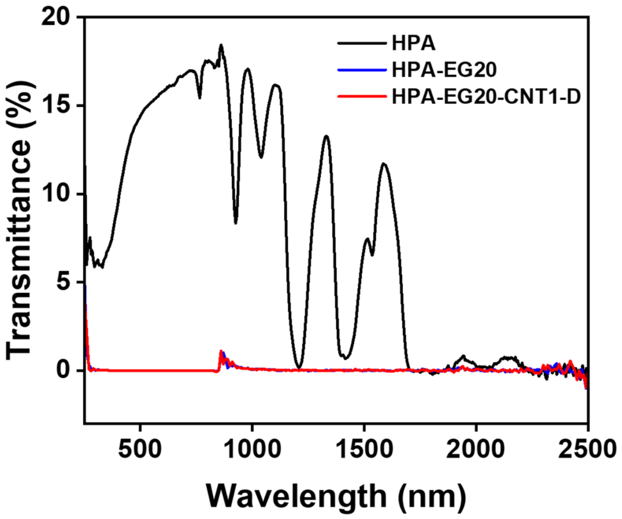

Figure S8. Transmission spectra of HPA, HPA-EG20 and HPA-EG20-CNT1-D composites.

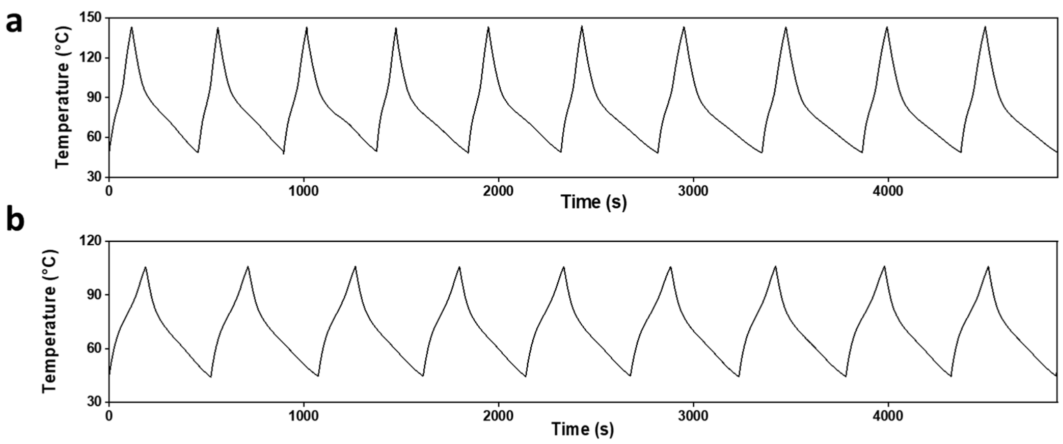

Figure S9. Recycled charging and discharging tests by placing the HPA-EG20-CNT1-D composites under direct concentrated solar illumination (**a**: 8 kW/m^2, **b**: 5 kW/m^2) followed by natural cooling.

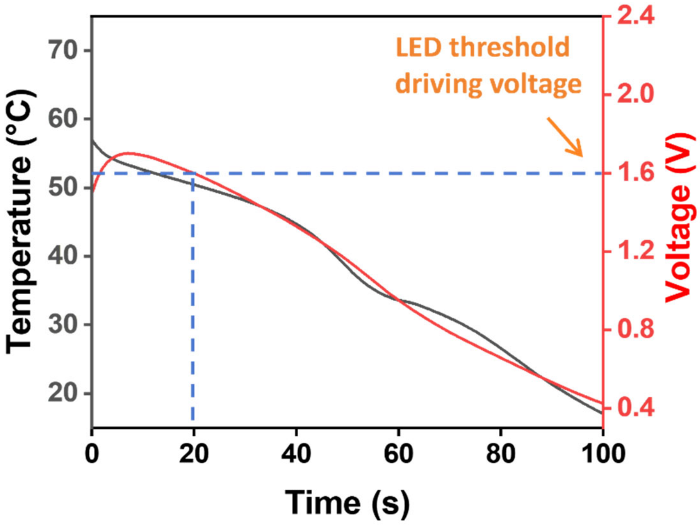

Figure S10. Temperature evolution profile and corresponding output voltage during discharging of PA-EG20 composites.
